# Parasites of pufferfish, *Lagocephalus* spp. and *Torquigener flavimaculosus* of the Israeli Mediterranean: A new case of Lessepsian endoparasites^☆^^☆☆^

**DOI:** 10.1016/j.ijppaw.2022.09.003

**Published:** 2022-10-19

**Authors:** Michael Gabel, Patrick Unger, Stefan Theisen, Harry Wilhelm Palm, Shevy Bat-Sheva Rothman, Nitzan Yitzhak, Arseniy R. Morov, Nir Stern

**Affiliations:** a Thünen Institute of Fisheries Ecology, Bremerhaven, Germany; b Faculty of Agricultural and Environmental Sciences Professorship for Aquaculture and Sea-Ranching (AQ), University of Rostock, Rostock, Germany; c Department of Zoology and The Steinhardt Museum of Natural History, Tel Aviv University, Tel Aviv, Israel; d Israel Oceanographic and Limnological Research Institute (IOLR), Haifa, Israel

**Keywords:** Lessepsian migration, Molecular identification, Morphology, Trypanorhyncha, Opistholobetines, Maculifer dayawanensis, Caligus fugu, Taeniacanthus lagocephali

## Abstract

With the opening of the Suez Canal as a link between the Red Sea and the Mediterranean Sea in 1869, the biogeographical event of the Lessepsian migration has been starting. Aided by beneficial conditions in the new habitat, almost 500 marine species have immigrated and often established themselves in the Mediterranean Sea, including several pufferfish species, with all of them extending their range and becoming important components of the local fauna. The parasitic fauna of these pufferfish has scarcely been examined in the Mediterranean Sea or in their native range, which provides the opportunity to study host-parasite interaction in a new habitat. The present study describes the parasitic fauna in four alien invasive pufferfish species (*Lagocephalus guentheri, L. sceleratus, L. suezensis*, and *Torquigener flavimaculosus*) of various sizes and ages on the Israeli Mediterranean coast. The parasite fauna of these species was diverse (*Maculifer dayawanensis* Digenea; *Calliterarhynchus gracilis*, *Nybelinia africana* and Tetraphyllidea larvae Cestoda; *Hysterothylacium reliquens*, *Hysterothylacium* sp. and *Raphidascaris* sp. Nematoda; *Trachellobdella lubrica* Hirudinea and *Caligus fugu* and *Taeniacanthus lagocephali* Copepoda) and consisted of mostly generalist species, most likely acquired in the new habitat, and specialist copepod ectoparasites, having co-invaded with the pufferfish. Additionally, the oioxenic opecoelid digenean *Maculifer dayawanensis* was found in two pufferfish species. The genus was previously only known from the Indo-Pacific Ocean, representing the eighth reported case of a Lessepsian endoparasite so far. Our results suggest a change in parasite fauna to native Mediterranean species in the pufferfish like previously reported in other Lessepsian migrant predatory fish species and a wider spread of co-invasion of fish endoparasites to the Mediterranean Sea than previously assumed. The study also provides several new host records and the first report for parasites in *T. flavimaculosus.*

## Highlights

• A comprehensive report of pufferfish parasites from Israeli Mediterranean Sea.
• Description of the parasitic fauna in four alien invasive pufferfish species on the Israeli Mediterranean coast.
• First report for parasites in *T. flavimaculosus*.
• Number of Lessepsian endoparasites is further increasing.
• First photographic record of Digenea from genus *Maculifer* Nicoll, 1915.

## Graphical abstract

**Figure:**
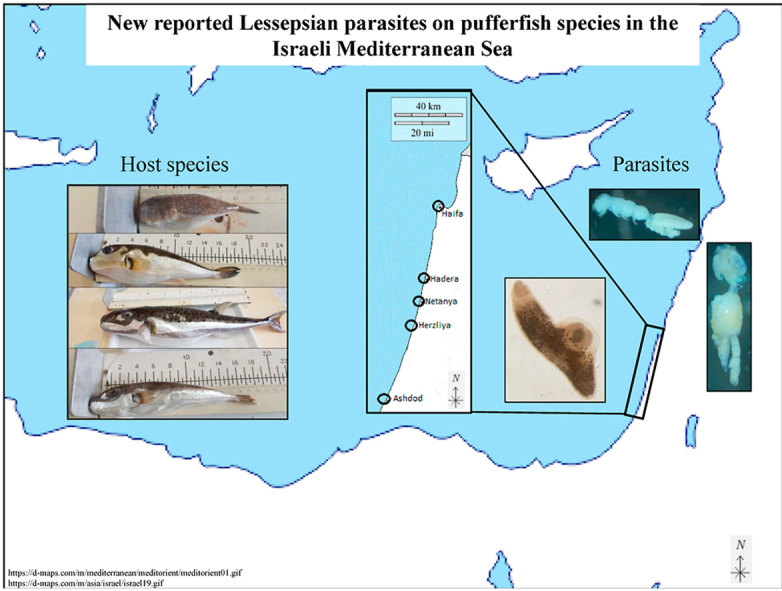

## 1 Introduction

The opening of the Suez Canal in 1869 brought down several million years of geographical isolation and enabled the immigration of Red Sea species to the Mediterranean Sea (Por, 1978; Galil et al., 2018), This ‘Lessepsian migration’ caused a dramatic change in the fauna of the Levantine Basin and, to a lesser degree, the Mediterranean Sea in general (Por, 1978; Galil, 2007a, b). By 2020, 400–500 species from the Red Sea could be confirmed in the Mediterranean Sea, and although the rate is slowing down compared to previous decades, the number is still growing (Zenetos et al., 2012; Galil et al., 2020). Among the alien invasive species are five pufferfish, which originally inhabited the Red Sea and the Indo-Pacific Ocean (Golani et al., 2021). The species are the diamond-back puffer (*Lagocephalus guentheri* Miranda Ribeiro, 1915) first reported from the Dodecanese Islands (Greece) (Sanzo, 1930), the Suez puffer (*Lagocephalus suezensis* E. Clark & Gohar, 1953) first reported from Lebanon (Mouneimne, 1977), the yellow-spotted puffer (*Torquigener flavimaculosus* Hardy & Randall, 1983), first reported in Israel (Golani, 1987), the silver-cheeked toadfish (*Lagocephalus sceleratus* (Gmelin, 1789)) reported simultaneously from Israel (Golani and Levy, 2005) and Turkey (Akyol et al., 2005) and the rare and diminutive bathydemersal spiny blaasop (*Tylerius spinosissimus* (Regan, 1908)), reported from the Island of Rhodes (Greece) (Corsini et al., 2005). All of the species have extended their range after introduction, in particular *L. sceleratus*, which is now considered both a massive threat and a significant nuisance to the local ecosystem and human population alike (Streftaris and Zenetos, 2006; Kalogirou et al., 2010, 2012; Nader et al., 2012; Kalogirou, 2013).

The knowledge on pufferfish parasites is mostly limited to reports of cultured fish from Japan (Ogawa and Inouye, 1997; Ogawa and Yokoyama, 1998) and the Hawaiian Islands in the Central Pacific (Palm and Bray, 2014). Reports however often focus more on the recorded parasites than their host (Ikeda et al., 2006; Ito et al., 2006; Mejia-Madrid and Aguirre-Macedo, 2011), and very few comprehensive studies on the parasite fauna of wild living species were done (Fajer-Ávila et al., 2004; Álvarez-Borrego and Fajer-Ávila, 2006; Beveridge et al., 2014; Prasad and Radhakrishnan, 2010). For the genus *Lagocephalus* Swainson, 1839 some comprehensive publications exist (e. g. El-Lamie and Abdel-Mawla, 2012; Prasad and Radhakrishnan, 2010; Bakopoulos et al., 2017), but on the species level, the inaccurate distinction between closely related and similar-looking pufferfish may complicate the issue on taxonomic status (Matsuura et al., 2011; Farrag et al., 2015; Giusti et al., 2019).

The partial loss (strip off) of the native specialized parasite fauna is considered part of the success of invasive species (enemy release hypothesis) (Torchin et al., 2002; Colautti et al., 2004; Hänfling, 2007; Vignon et al., 2009; MacColl and Chapman, 2010). Many factors, however, contribute to the success of Lessepsian migrant fish (Arndt and Schembri, 2015), which also tend to accumulate a new, rich generalist parasite fauna in their newly colonized habitats (Merella et al., 2016; Boussellaa et al., 2018). The Lessepsian migration of parasites has been recorded first in 1971/1972 for ectoparasites on fish. Both monogeneans on rabbitfish (Siganidae) (Paperna, 1972; Ktari and Ktari, 1974) and copepods on grey mullets (Mugilidae) (Botros, 1971) have been reported to live on their native hosts in the newly colonized habitat, thus having survived the adverse salinity, oxygen, and temperature conditions (Por, 1978) of the Suez Canal. Findings of ectoparasitic isopods (Trilles and Bariche, 2006), monogeneans (Pasternak et al., 2007), and copepods (El-Rashidy and Boxshall, 2009) immigrating with their hosts have increased in later years. Unlike ectoparasites, Lessepsian endoparasites are protected inside their hosts from adverse external conditions during the passage of the Suez Canal. Because of their heteroxenous life cycles, they may lack the obligatory intermediate hosts to close them in the new habitat and thus have a lower chance of establishing themselves. So far, three species of Myxozoan parasites have immigrated and established from the Red Sea (Diamant, 2010), and four digenean species have been reported (Fischthal, 1980; Merella et al., 2016). The most recent reports are *Allolepidapedon fistulariae* Yamaguti, 1940 and *Neoallolepidapedon hawaiiense* Yamaguti, 1965, are parasitizing in the blue-spotted cornetfish *Fistularia commersonii* Rüppell, 1838, another Lessepsian migrant fish. Both parasite species, well known from their host in the native habitat, so far however lack reports of larval stages in the Mediterranean Sea and may have simply co-invaded as adults with individual fish from the Red Sea or may just be at a starting point of an establishment (Pais et al., 2007; Merella et al., 2010, 2016). The other two reported species are an old single report of *Thulinia microrchis* (Yamaguti, 1934) Bray, Cribb & Barker***,*** 1993 in the marbled spinefoot *Siganus rivulatus* Forsskål & Niebuhr, 1775 and the taxon inquirendum larval form *‘Monilicaecum ventricosum’* Yamaguti, 1942 in the brushtooth lizardfish *Saurida undosquamis* (J. Richardson, 1848) (Fischthal, 1980). None of these four digeneans have been found in Lessepsian pufferfish or any native Mediterranean fish species.

Three studies exist on pufferfish parasites from the Mediterranean Sea and Suez Canal (El-Lamie and Abdel-Mawla, 2012; Özak et al., 2012; Bakopoulos et al., 2017). At present, of the Lessepsian migrant species, only *L. sceleratus* has a comprehensive report of parasites from both the native and invaded range. For *L.* *guentheri*, reliable data is only available from the Mediterranean Sea and the Suez Canal (El-Lamie and Abdel-Mawla, 2012; Özak et al., 2012). In the case of *L. suezensis*, the only reported parasite is the invasive caligid copepod in the Mediterranean Sea (Özak et al., 2012) (Overview in Table 1). For *T. flavimaculosus*, no reports exist at all. This present study aims to describe and provide new information on the parasite fauna of the Mediterranean invasive pufferfish by examining *L. guentheri*, *L. sceleratus*, *L. suezensis*, and *T. flavimaculosus*. It also aims to provide the first report for parasites in *T. flavimaculosus* throughout its distribution. The qualitative and quantitative assemblage of pufferfish may finally provide new insights into their role as possible carriers of potentially dangerous invasive parasite species.

**Table 1:** Previously reported parasites of the Lessepsian pufferfish in their natural range and the Mediterranean Sea.

Host/Parasite	Region (a)	Prevalence (b)	Literature (c)
*Lagocephalus guentheri*			
Cestoda			
Tetraphyllidea (plerocercoid)	SUE	6.1% (294)	1
Nematoda			
*Cucullanus* sp. (larva)	SUE	4.1% (294)	1
Copepoda			
*Caligus fugu*	MES	80.0% (35)	2
*“*	SUE	34.6% (294)	1
*Taeniacanthus lagocephali*	MES	94.0% (50)	2
*“*	SUE	34.6% (294)	1
*Lagocephalus sceleratus*			
Monogenea			
*Heterobothrium tonkinense*	SCS	x	3
*“*	GOT	x	4
Digenea			
*Bianium arabicum*	NEC	x	5
*Zoogonoides viviparus*	NEC	x	6
*Maculifer subaequiporus*	SEY	x	7
Cestoda			
*Dasyrhynchus basipunctatus* (plerocercoid)	NEC	x	8
*Nybelinia indica* (plerocercoid)	NEC	x	8
Nematoda			
*Anisakis* sp. (larva)	MES	4.9% (41)	9
*Hysterothylacium aduncum*	MES	14.6% (41)	9
*Philometra lagocephali*	NEC	50.0% (2)	10
*Philometra tenuicauda*	NEC	25.0% (8)	11
Copepoda			
*Caligus rufimaculatus*	SAF	x	12
*Taeniacanthus kitamakura*	NEC	x	13
Isopoda			
*Gnathia* sp*.*	MES	2.4% (41)	9
*Lagocephalus suezensis*			
Copepoda			2
*Caligus fugu*	MES	34.7% (23)	

a MES Mediterranean Sea, SUE Suez Canal, NEC New Caledonia, SEY Seychelles, SCS South China Sea, GOT Gulf of Tonkin, SAF South Africa.

b For each source and locality, the simple presence (“X”) reported or the prevalence (”%“) of the parasite. The sample size given in brackets.

c 1 El-Lamie and Abdel-Mawla (2012); 2 Özak et al. (2012); 3 Jianying et al. (2003); 4 Hung et al. (2020); 5 Quilichini et al. (2015); 6 Bray and Justine (2014); 7 Toman (1992); 8 Beveridge et al. (2014); 9 Bakopoulos et al. (2017); 10 Moravec and Justine (2008); 11 Moravec and Justine (2009); 12 Hayes et al. (2021); 13 Justine (2010).

## 2 Materials and methods

### 2.1 Study area and data collection

All the fish examined in this study were collected between the 3^rd^ of June 2019 and the 1^st^ of June 2020 (Table 3). The samplings were carried out by multiple methods (gill nets, trawl nets, fishing rods, strand findings) with the aid of anglers, artisanal and commercial fishermen, in depths of 1–80 m over sandy and rocky bottoms. The collected fish were either immediately dissected, kept at 4 °C for a maximum of 48 h, or frozen at −20 °C until dissection at the ichthyological laboratory at the Israeli Oceanographic and Limnological Research Institute (IOLR).

**Table 2:** PCR protocols and primers used for molecular identification of the parasites.

Group	Primer F	Primer R	Region	Initial Denaturation (° C/min)	Denaturation (° C/min)	Annealing (° C/min)	Elongation (°C/min)	Cycles	Termination (°C/min)
Nematoda	TK1	NC2	ITS1-5.8s-ITS2	95/1	94/0.75	55/0.75	72/0.75	40	72/10
Cestoda	300F	ECD2	28S rRNA	94/4	94/0.5	52/0.5	72/1	40	72/7

**Table 3:** Catch data, location, and measurements of the examined specimens of the four pufferfish species at the Israeli Mediterranean coast.

N	Locality	Date	SUEZ (a) L_T_ (cm)/W_T_ (g) (n) (b)	SCEL (a) L_T_ (cm)/W_T_ (g) (n)	GUEN (a) L_T_ (cm)/W_T_ (g) (n)	FLAVI (a) L_T_ (cm)/W_T_ (g) (n)
1	Ashdod	03/06/2019	–	–	–	8.2–11.1/9.6–24.1 (18)
2	Ashdod	12/09/2019	–	36.0–58.5/501.7–2612.5 (22)	–	–
3	Ashdod	05/12/2019	14.8–19.2/43.2–96.6 (5)	–	9.7–37.0/15.3–908.5 (17)	7.5/8.7 (1)
4	Netanya	07/02/2020	9.5–19.9/10.7–94.0 (30)	–	–	7.8–11.7/9.2–27.3 (16)
5	Herzliya/Netanya	19/02/2020	–	20.5–31.0/104.4–313.4 (4)	17.5–23.1/94.1–187.3 (2)	–
6	Haifa	23/03/2020	–	45.0–45.5/978.2–1007.4 (2)	–	–
7	Haifa	28/04/2020	–	64/2861.3 (1)	–	–
8	Netanya/Hadera	04/05/2020	–	28.0/224.3 (1)	16.2–19.5/100.9–121.5 (4)	–
9	Haifa	08/05/2020	–	52.5/1486.0 (1)	–	–
10	Haifa	30/05/2020	–	61.5/2734.0 (1)	–	–
11	Ashdod	01/06/2020	–	30.5–54.3/299.3–1849.7 (3)	18.8–43.0/122.7–1125.8 (11)	–

a SUEZ Lagocephalus suezensis; SCEL L. sceleratus; GUEN L. guentheri; FLAVI Torquigener flavimaculosus.

b *L*_*T*_ total length; *W*_*T*_ total weight.

### 2.2 Studied species and dissection

A total of 139 specimens were examined (n = 35 specimens of each *Lagocephalus sceleratus, L. suezensis*, and *Torquigener flavimaculosus*, n = 34 specimens of *L. guentheri*). Dissections were performed following standard procedures (Cribb and Bray, 2010; Palm and Bray, 2014; Klimpel et al., 2019). First, basic morphometric data, total length (L_T_), standard length (L_S_), and total weight (W_T_) were taken. Hereafter, the body surface and openings (eyes, skin, fins, gills, nostrils, anus, and mouth cavity) were investigated macroscopically. For dissection, gill arches and eyes were removed first, afterwards, the body cavity was opened and the internal organs (liver, gall bladder, digestive tract, gonads, swim bladder, and heart) were removed. The organs were opened and inspected macroscopically and with a stereomicroscope (Zeiss Stemi 508 0.63–5.0, Carl Zeiss Microscopy GmbH, Jena, Germany). All found parasites were mechanicaly cleaned of stomach content and transferred into buffered saline solution (0.9% NaCl) and morphologically gross sorted. Parasites were transferred and stored in 96% EtOH for molecular identification or 80% EtOH for morphology. The stomach content was also identified to the lowest possible taxonomic level.

### 2.3 Mounting

Parasites were either cleared and mounted in glycerine (after Klimpel et al., 2019) and sealed with paraffin or Aceto-Carmine stained and mounted in Canada balsam (after Palm, 2004). Parasites were identified using a light microscope (OLYMPUS BX53), camera (OLYMPUS DP74) and photo software (OLYMPUS cellSens Dimension 1.6 (Olympus Corporation, Shinjuku, Tokyo, Japan), and assorted literature (Pearse, 1952; Deardorff and Overstreet, 1981; Dojiri and Cressey, 1987; Moravec, 1998; Euzet, 1994; Palm, 2004; Boxshall and El-Rashidy, 2009; Ben Ahmed et al., 2015; Martin et al., 2018; Guardone et al., 2020; Noël and Sittler, 2021).

### 2.4 Molecular identification of cestodes and nematodes

Total genomic DNA was extracted using a Qiagen® DNeasy Blood & Tissue Kit (QIAGEN GmbH, Hilden, Germany) following the manufacturer's protocol. In the case of very high parasite counts, a representative subsample, as described in Santos at al. (2017), was analysed. Extracted DNA was used to amplify the ITS1-5.8s-ITS2 sequence region (for nematodes) and the 28S rRNA gene (for cestodes) For PCR protocols see Table 2. The samples were purified with Qiagen QIAqick® PCR Purification Kit (QIAGEN GmbH, Hilden, Germany) and sent to *SEQLAB* (Microsynth SEQLAB GmbH, Göttingen, Germany) for sequencing. The obtained sequences were edited with MEGA X ((Kumar et al., 2018) and DNA Baser V4 Software (Heracle BioSoft SRL, Arges, Romania). The results were compared to the GenBank™ gene database and selected sequences were additionally uploaded to the GenBank™ for reference. Voucher specimens were deposited in the ‘Zoologische Lehrsammlung’ in Berlin, Germany, for further research.

### 2.5 Statistical analyses

The prevalence (P), mean intensity (mI), intensity (I), and abundance (A) of each parasite species were calculated, according to Bush et al. (1997). The parasite community composition was calculated by an abundance-based altered core-/satellite-species concept as used by Zander et al. (2000). The ratio of ecto-versus endoparasites (E/E) was calculated according to Palm and Bray (2014). Differences in parasite communities between the pufferfish species were compared by creating a Bray-Curtis similarity measure with PRIMER 7 software (Primer-e, Massey University, Albany, Auckland, New Zealand) and running ANOSIM (Analysis of Similarities), SIMPER (Analysis of Similarity Percentages) analysis and an MDS (nonmetric multi-dimensional scaling plot).

## 3 Results

### 3.1 Morphometric fish measurements

The morphometric data for the species varied considerably between *Lagocephalus guentheri* (9.7–43 ($\bar{X}$: 23.64) cm L_T_, 15.29–1125.80 ($\bar{X}$: 296.39) g W_T_), *L. sceleratus* (20.5–64.0 ($\bar{X}$: 43.09) cm L_T_, 104.41–2861.30 ($\bar{X}$: 1030.58) g W_T_), *L. suezensis* (9.5–19.5 ($\bar{X}$: 14.30) cm L_T_; 10.72–94.0 ($\bar{X}$: 36.83) g W_T_) and *Torquigener flavimaculosus* (7.5–11.7 ($\bar{X}$: 9.75) cm L_T_; 8.70–24.34 ($\bar{X}$: 17.55) g W_T_). In the two large species, the mean for L_T_ and W_T_ was considerably higher than even the biggest specimens of the two smaller species (Table 3).

### 3.2 Types and prevalence of parasites

The parasitological analyses of the 139 pufferfish retrieved ten species of parasites and an additional protozoan parasite. The parasites were identified to the lowest possible taxonomic level (Table 3). The metazoan parasite groups/taxa were Digenea (1), Cestoda (3), Nematoda (3), Hirudinea (1), and Copepoda (2).

**Fig. 1:**
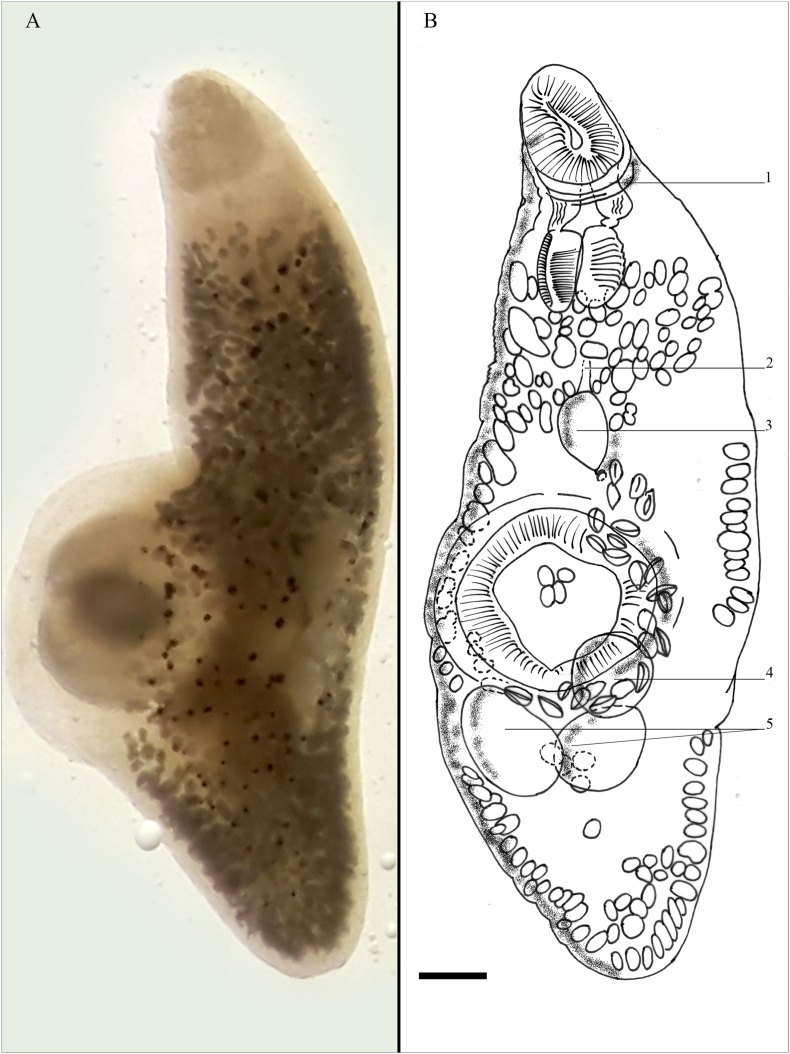
Habitus of alive (A) and mounted line-drawn (B) specimens of *Maculifer dayawensis* from the intestinal tract of *Lagocephalus guentheri*, including all visible characteristics. The black spots in the alive specimen – the pigment granules. Diagnostic characteristics of the parasite - the muscular post-oral ring (1), genital pore (2), cirrus sack (3), ovary dorsally behind left testes (4) and opposite testes (5), as well as vitelline follicles reaching up to the pharynx. Follicles extend over the whole body and are partially omitted in the drawing for the sake of clarity.

The adult Digenea were found in the digestive tract lumen of *L. guentheri* and *T. flavimaculosus*, with a prevalence and intensity of 11.8% and 1–9 for *L. guentheri* and 2.9% and 1 for *T. flavimaculosus*. Total length (L_T_) of Digenean (n = 2) was 1772–2225 μm, body width 895–915 μm, but when alive, distinctly more elongated (L_T_ > 2x body width). The oral sucker was subterminal, a diameter of 110–251 μm. The following prepharynx was surrounded by a prominent, muscular post-oral ring. The pharynx length x width was 123 μm × 110 μm. The ventral sucker was closely located to mid-body and larger than the oral sucker, with a diameter of 290–425 μm. Vitelline follicles measured 22 μm × 21 μm in diameter, reaching from the pharynx to the end posterior of the body cavity. Pigment granules were present, and sparsely distributed throughout the body. Testes were located opposite each other, measuring 160 μm × 114 μm and 146 μm × 116 μm. The ovary overlapped the right testis dorsally and was located posterodorsally to the ventral sucker. Eggs were oval and operculated, length x width was 26–43 μm x 14–30 μm (Fig. 1). The presence of the muscular post-oral ring around the prepharynx, body length over 2x body width, and sparse pigment vesicles throughout the body identified the Digenea as genus *Maculifer* Nicoll, 1915 according to Martin et al. (2018). The body size, size, and position of the measured internal organs either pointed towards *M. indicus* or *M. dayawanensis* since the exact position of the genital pore in relation to the intestinal bifurcation was not recognizable. The extent of the vitelline follicles from the pharynx, not just from the intestinal furcation to the posterior end of the body and its hosts identified as *Maculifer dayawanensis* Sheng and Tong, 1990 according to Sheng and Tong (1990) and Madhavi and Bray (2018). Specimens are deposited in the collection of the Museum für Naturkunde Berlin (catalogue nos. ZMB E.7672; ZMB E.7673).

All three found cestode species were plerocercoid larval stages from two families, Trypanorhyncha and Tetraphyllidea. Two species of Trypanorhyncha were encysted on various organs, the Tetraphyllidea were free inside the digestive system. The first species was found encysted on the intestines (mesenteries, swim bladder) surrounded by connective tissue capsules in the body cavity in *L. sceleratus*. Prevalence was 22.9% and intensity 1–3. Freed from the blastocysts (n = 3), L_T_ was 3.4–10.2 mm with a transparent long and slender body (width 618.7–1000.8 μm at pars bothrialis). Scolex is long, slender, and well-developed. Two opposing posteriorly slightly notched bothridia are with length of 529.1–839.0 μm. Four long tentacles (length 1081.4–1934.5 μm; basal width 25.2–63.7 μm) are bearing hollow hooks. Tentacle sheaths are as spirals inside the scolex, ending in four elongated (length 484.6–1934.5 μm; width 131.9–224.4 μm) bulbs. The appendix behind the pars bulbosa is long and slender. All measurements and general morphological traits were consistent with the description by Palm (2004). The morphology, morphometric measures, and shape of the tentacle sheaths identified the specimens as plerocerci of *Callitetrarhynchus gracilis.* Blasting on the GenBank against 28S rRNA sequence (n = 1, accession no. OM867573) also gave 100% Query Cover and 98.06–100% Identity for *C. gracilis* (accession nos. FJ572957.1; DQ642758.1-DQ642759.1; MG694210.1; MN488532.1)*.*

The second species was encysted on the stomach wall of *L. guentheri.* P was 2.9% and intensity was one individual. L_T_ was 766.0 μm, width 457.6 μm at pars bothrialis. A compact, well-developed scolex with four opposing triangular bothridia (length 379.5 μm). Four straight and short tentacles with solid hooks (length 272.8 μm; basal width 29.7 μm), retracted in straight sheathes, ending in four short bulbs (length 231.8 μm). Appendix behind the pars bulbosa short. The measurements fitted the ones by Palm (2004) for plerocercoids of *Nybelinia africana* Dollfus, 1960. The small size, morphological features, and morphometric measurements identified it as a plerocercoid of *N. africana.*

The third cestode were plerocercoids in the digestive tracts of *Lagocephalus* spp. Prevalence and intensity ranged from 11.4% and from one to nine individuals in *L. suezensis* to 50.0% and from one to 200 individuals in *L. guentheri.* L_T_ was 0.2–2 mm, with the bodies being transparent and brittle, showing a diverse body shape, with several distinct morphotypes. Four bothria were present on the scolex, sometimes sitting on peduncles, sometimes retracted into the scolex or completely absent and a central apical sucker was present on the very top of the scolex. The morphological features were identical to the characteristics described in Euzet (1994) and used for his diagnosis of Tetraphyllidea Carus, 1863. The four bothria in combination with the apical sucker and the location of the plerocercoids attributed the cestodes to the order Tetraphyllidea.

Among the nematodes, three species were present. The first species was present in all four pufferfish species, living freely inside the intestine. Only two specimens were found enrolled on the liver and gills in *L. suezensis*. Prevalence and intensity ranged from 31.4% and from one to eleven individuals in *L. suezensis* to 88.6% and from one to 80 parasites in *L. sceleratus.* Larval stages (L3/L4) were present in all pufferfish, adults only in larger specimens of *L. sceleratus* and only a single adult male in *L. guentheri.* L_T_ was 2–40 mm, with a long slender body reaching its greatest width near the middle. The cuticle was smooth, with no ornaments on the conical tail. Both posteriorly directed ventricular appendix and anteriorly directed internal caecum were present. Adults with three pronounced lips and spicules (males), or visible ovaries (females). Measurements and morphological features fell within the description of Deardorff and Overstreet (1981) provided for *Hysterothylacium reliquens* (Norris & Overstreet, 1975) Deardorff and Overstreet, 1981. Blasting on the GenBank™ against ITS1-5.8s-ITS2 sequence regions (n = 5, accession nos. OM888581- OM888585) resulted in a 94–100% Query Cover and 99.8–100% Identity for deposited vouchers of *H. reliquens* from the Mediterranean Sea (accession nos. MF062507.1-MF062509.1), Arabian Gulf (accession nos. KX786286.1-KX786293.1) and the Atlantic US-coast (accession nos. MF668856.1; MF668873.1; MF668876.1; MF668880.1) and confirmed the identity of *H. reliquens*.

The second nematode was indistinguishable from *H. reliquens* larvae and could only be identified through molecular identification after blasting. The host was *L. guentheri*, with a prevalence of 5.8% and an intensity of two individuals. Only two specimens could be identified (accession nos. OM888586- OM888587). Query Cover was 87–88% and identity 100% for an unidentified larval form of the genus *Hysterothylacium* Ward & Magath, 1917 from the Mediterranean Sea (accession nos. MT365529.1-MT365537.1) described by Guardone et al. (2020).

The third nematode was only present in *L. sceleratus*, with a prevalence of 2.9% and intensity of two parasites, with two specimens found. All measured specimens were incomplete, so no L_T_ was measurable. The body of the nematode was very elongated and slender, reaching the greatest width near the middle. A posteriorly directed ventricular appendix was present, and intestinal caecum was absent. Length of ventricle was 136–162 μm, width of ventricle was 97–100.6 μm, and oesophagus length was 1488–2009 μm. Three prominent hooked lips were located on the head. One measured specimen was an adult female, having fully developed ovaries and a gonopore. The shape of the lips, the presence of a posteriorly directed ventricular appendix, the absence of an intestinal caecum, and the measurements defined the parasite as belonging to the genus *Raphidascaris* Railliet & Henry, 1915 as described by Moravec (1998). Specimen are stored in the institute's collection.

The single Hirudinea specimen was found on the skin of *L. sceleratus* under the pectoral fin, with a prevalence of 2.9% and an intensity of one individual. L_T_ was 3.5 cm and the body was of brown-green color. The anterior part of the body was distinctly slenderer and of a contrasting yellow in color. On the oral sucker was one pair of eyes. The body greatly widened towards the rear third, at the location of a pair of contractive vesicles, and then tapered down into a small posterior sucker without ocelli. The skin was smooth, except for 14 pairs of contracting lateral vacuoles on the side of the body. The size, presence of vesicles and vacuoles, coloration and present morphological features distinguished the leech as *Trachelobdella lubrica* (Grube, 1840) according to the description given by Ben Ahmed et al. (2015) and Noël and Sittler (2021).

Two copepod species were free-living on the gill filaments of the pufferfish. The first copepod was present on *Lagocephalus* spp. Prevalence and intensity ranged from 20.0% and from one to two individuals in *L. suezensis* to 38.2% and from one to eight individuals in *L. guentheri*. Mostly adults and rarely chalimus stages were present. The genus could be identified as *Caligus* Müller, 1785 by the presence of two suction organs (lunnules) on the base of the antennas in combination with a visible single nauplius eye on top of the cephalosome. Measurements for adult ♀ (n = 4) were: 3.1–3.5 mm for L_T_, cephalothorax length x width 1.0–1.3 mm x 1–1.2 mm. The length of the genital complex was 1.3–1.6 mm, 1.36–1.90 times as long as the two-segmented abdomen (0.7–1.0 mm), while the first segment of the abdomen was 2.11–2.59 times as long as the second. The maxillipeds showed a well-developed, tapering process proximally on the medial margin. The measurements, traits, and hosts are consistent with the description for *Caligus fugu* Yamaguti and Yamasu, 1959 by Boxshall and El-Rashidy (2009).

The second copepod exclusively inhabited *L. guentheri.* Prevalence was 88.2% and intensity was from one to 42 individuals. Egg-bearing females were predominantly present, whereas males were significantly fewer. Measurements for ♀ (n = 2) were: L_T_ 2.3–2.7 mm, cephalothorax length x width 0.5–0.7 mm x 0.7–0.8 mm, 22–26% of L_T_. Body three-segmented, with segments almost as wide as cephalothorax, abdomen with four segments. Caudal ramus length x width 66–74 μm × 40 μm, 1.7–1.9 times longer than wide. Four rows of spinulae on the dorsoventral surface of the genital segment, with a fifth close to the base of the caudal ramus. The diagnosis for *Taeniacanthus lagocephali* Pearse, 1952 by Dojiri and Cressey (1987) fitted the measured specimens. The measurements, distinct three free thoracic segments equal in width to the cephalothorax, armature at the anal segment, and its presence solely on *L. guentheri*, a close relative of the originally described hosts, confirmed the species as *T. lagocephali.*

In Table 4 the quantitative parasite data of the four pufferfish species from the Israeli Mediterranean Coast is provided. Regarding the communities, four parasite species could be classified as common (core species) with an abundance of >2.0 in at least one of the pufferfish species, one species was secondary, with an abundance of 0.6–2, two species were satellite species with an abundance of 0.2–0.6 and four were rare species with an abundance of <0.2 (Tab 4). The parasite assemblages were dominated by *H. reliquens (*P: 3.43–88.57%, mI: 3.00–17.68), parasitic Protozoa (P = 5.71–51.43%), Tetraphyllidea (P = 11.43–50.00%, mI = 2.75–45.12), *Caligus fugu* (P = 20.00–38.24%, mI = 1.14–2.34) and *T. lagocephali* (P = 88.24%, mI = 7.73).

**Table 4:** Found parasites of *Lagocephalu*s spp. & *Torquigener flavimaculosus* from the Israeli Mediterranean Sea.

Parasite	Location	GUEN (a)			SCEL (a)			SUEZ (a)			FLAVI (a)		
		P (%) (b)	I (mI) (b)	A (b)	P (%)	I (mI)	A	P (%)	I (mI)	A	P (%)	I (mI)	A
Protozoa^a^	Mesenteries, intestines, eyes	20.6	–	29.4	51.4	–	28.6	34.3	–	28.6	5.7	–	28.6
Digenea													
*Maculifer dayawanensis*^b^	Digestive tract	11.8	1-9 (3)	0.4							2.9	1 (1)	0.03
Cestoda													
*Calliterarhynchus gracilis* (plerocercus)	Intestines (cyst)				22.9	1-3 (2)	0.5						
*Nybelinia africana* (plerocercoid)	Stomach wall (cyst)	2.9	1 (1)	0.03									
Tetraphyllidea (plerocercoid)^a^	Digestive tract	50.0	1-200 (45.1)	22.6	17.1	3-63 (17.8)	3.1	11.4	1-9 (2.8)	0.3			
Nematoda													
*Hysterothylacium reliquens* (L3-L4, adult)^a^	Digestive tract, intestines	67.7	1-18 (3.9)	2.6	88.6	1-80 (17.7)	15.2	31.4	1-11 (3)	0.9	34.4	1-23 (4.6)	1.6
*Hysterothylacium* sp. (L3)	Digestive tract	5.8	1 (1)	0.1									
*Raphidascaris* sp. (adult)	Digestive tract				2.9	2 (2)	0.1						
Hirudinea													
*Trachellobdella lubrica*	Surface				2.9	1 (1)	0.03						
Copepoda													
*Caligus fugu*^b^	Gill filaments	38.2	1-8 (1.8)	0.7	20.0	1-5 (2.4)	0.5	20.0	1-2 (1.1)	0.2			
*Taeniacanthus lagocephali*^ab^	Gill filaments	88.2	1-42 (7.7)	6.8									

a GUEN Lagocephalus guentheri; SCEL L. sceleratus; SUEZ L. suezensis; FLAVI Torquigener flavimaculosus.

b P prevalence %, I intensity, mI mean intensity (range in brackets), A abundance, frequently occurring parasites (core species) are mentioned with superscript a, Lessepsian parasites with superscript b.

All four pufferfish species were infected with less ecto-than endoparasites. The E/E ratio for *Torquigener flavimaculosus*, which had no ectoparasites at all, was 0 (0/2). For *Lagocephalus guentheri*, it reached 0.5 (2/4), for *L. sceleratus* 0.67 (2/3), and for *L. suezensis* 1.0 (1/1). For all species combined, the E/E ratio was 0.50 (3/6).

### 3.3 Differences in parasite assemblages

**Fig. 2:**
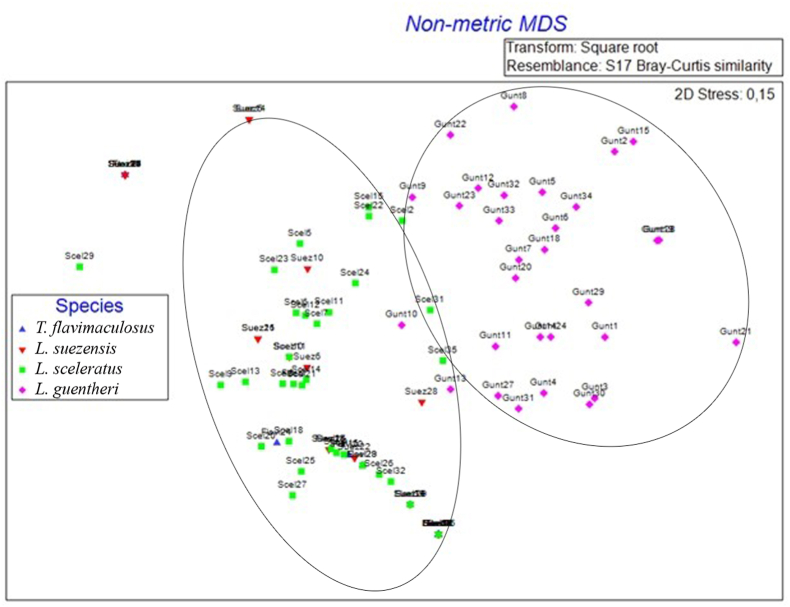
Multidimensional scaling (MDS) plot displaying the similarity of the parasite fauna of the four pufferfish species. Only infected pufferfish shown and one extreme outlier of *Torquigener flavimaculosus* excluded for a more compact display.

The differences in parasite assemblage between the pufferfish species were mostly determined by the most common and abundant parasites (*H. reliquens*, Tetraphyllidea, protists, and *T. lagocephali*). ANOSIM analysis showed that the parasite assemblies of each of the four species differed from the others, but with some overlaps for each species (R = 0.48; p = 0.001–0.015). SIMPER analysis showed that assemblies were the most similar between *T. flavimaculosus* and *L. sceleratus* (Average dissimilarity = 61.4%), followed by *T. flavimaculosus* and *L. suezensis* (70.1%) being equally different from *L. suezensis* and *L. sceleratus* (70.3%). The differences between *L. scleratus* and *L. guenteri* were larger (79.5%) and the biggest dissimilarity was found between *T. flavimaculosus* and *L. guentheri* (83.7%) as well as *L. suezensis* and *L. guentheri* (84.5%). Other factors such as size, size classes, or catch date failed to yield significant differences. An MDS plot (Fig. 2) also supported the results by showing a clustering in dependence of species with a sufficiently low-stress level (0.15). All infected *L.* *guentheri* formed a distinct cluster, as well as *L. sceleratus. L. suenzensis* clustered partially with *L. sceleratus*, whereas *T. flavimaculosus* showed the biggest overlap with *L. suezensis*.

### 3.4 Stomach content analysis

The stomach content of all four pufferfish species contained remains of a variety of invertebrate groups. The stomachs of *Lagocephalus guentheri* contained unidentifiable cephalopods, fish bones and otoliths, polychaet mandibles and ostracods. Identifiable prey items were headshield slug (*Philine* sp.), squids (*Loligo* sp.), cuttlefish (*Sepia officinalis*), swim crabs (*Charybdis* sp.), crucifix crab (*Charybdis feriata*), Blue swim crab (*Portunus segnis*) and mantis shrimp (*Erugosquilla massavensis*). Identified fish prey was two species of goatfish (*Upeneus pori, U. moluccensis*), pony fish (*Equulites klunzingeri*), striped eel catfish (*Plotosus lineatus*) and puffer fish (*Torquigener flavimaculosus)*. In *L.* *sceleratus,* parts of unidentifiable cephalopods, fish bones and otoliths, snail and mussel shells and marine isopods were present. Identifiable prey were cuttlefish (*S. officinalis*), swim crabs (*Charybdis* sp.), crucifix crab (*C. feriata*), lesser swimming crab (*Charybdis longicollis*) Blue swim crab (*P. segnis*), pebble crab (*Myra subgranulata*), box crab (*Arcania brevifrons*) and mantis shrimp (*E. massavensis*). Fish prey was striped mullet (*Mugil cephalus*), seabreams (*Pagellus* sp.), Goldband goatfish (*U. moluccensis*), pony fish (*E. klunzingeri*), reticulated file fish (*Stephanolepis diaspros*) and puffer fish (*T. flavimaculosus*). The stomachs of *L. suezensis* contained fragments of sea urchins, parts of unidentifiable cephalopod, prawns, gastropods, diatoms, crabs, mussel shells and tube worms. Prey items that could be identified were the snail *Cerithium scabridum* and swim crabs (*Charybdis* sp.). In *T. flavimaculosus*, unidentifiable gastropod and mussel shells, fragments of sea urchins, ascidia, crustacea and diatoms were present and the only identifiable prey was a snail (*C. scabridum*).

## 4 Discussion

The study was focused on parasites of the invasive pufferfish from Israeli Mediterranena Sea, with emphasis on potential Lessepsian migrant parasites. The first report of parasites in *Torquigener flavimaculosus* was additionally presented. Six out of seven newly reported Mediterranean native parasites represented new host records for the studied pufferfish. The opecoelid Digenea *Maculifer dayawanensis*, previously only recorded from the South-Chinese Sea, could be reported as a new Lessepsian endoparasite, found in the digestive tract of *Lagocephalus guentheri* and *T. flavimaculosus*. In addition, we confirmed the two previously reported Lessepsian copepods.

### 4.1 Digenea

The opecoelid Digenea *Maculifer dayawanensis* was either a rare or a satellite parasite found in the pufferfish, with a prevalence (2.9–11.8%) similar to previous reports of Indo-pacific pufferfish (11.0%) (Martin et al., 2018). Only mature specimens were present in *L. guentheri* and *T. flavimaculosus*. This record represents the fifth report of a macroscopic Lessepsian endoparasite from the Mediterranean Sea. All previous reports were also about Digenea in fish, have been found exclusively as adults or could not be properly identified (invalid larval stages) (Fischthal, 1980; Merella et al., 2010, 2016). In the case of the adult Digenea, a possible co-invasion with the hosts from the Red Sea could not be excluded (Pais et al., 2007; Merella et al., 2010, 2016). For the genus *Maculifer*, only adults have been reported from the Indo-Pacific (Nicoll, 1915; Cribb, 2005; Liu et al., 2010; Martin et al., 2018). Since *M. dayawanensis* was both present in the non-migratory *T. flavimaculosus* and the more migratory and habitat-changing *L. guentheri*, it is so far the strongest example of a Lessepsian endoparasite successfully establishing itself. The parasite was not found by El-Lamie and Abdel-Mawla (2012), who dissected 294 specimens of *L. guentheri*. Accordingly, *M. dayawanensis* either may have entered the Mediterranean Sea after 2012 or at an unknown point in the past, most likely coinciding with the immigration of *L. guentheri* in 1930 (Sanzo, 1930). Another possible introduction pathway is ballast water of ships instead of direct immigration, given the extremely long distance between the first reported location, Daya Bay in the South-Chinese Sea (Sheng and Tong, 1990), and the Israeli Mediterranean Sea. The Suez Canal is a major global shipping corridor, and rules for ballast water treatment only were implemented in 2017 (Galil et al., 2018). Ballast sediments in particular have not been studied as thoroughly as ballast water, are not always filtered, and may provide a suitable habitat for the first hosts, infected snails, releasing them during ballast water change (Gollasch et al., 2019). At least one opecoelid Digenea is reported to have a shortened life cycle, omitting intermediate and even final hosts in its ontogenesis and leaving the primary intermediate host already as adults (Barger and Esch, 2000). Both infected pufferfish species consumed gastropods, which are possible first intermediate hosts for *Maculifer* spp. A possible shortened life cycle would also explain the absence of encysted larval stages in the pufferfish stomachs from ingestion, as described for a closely related genus of Digenea (Cable, 1956). This study provides *L. guentheri* and *T. flavimaculosus* as new host records for *M. dayawanensis*.

### 4.2 Copepoda

The copepod *Caligus fugu* was among either the secondary or satellite parasites, and the presence of egg-carrying females and chalimus stages proved a successful reproduction in Israeli waters. This parasitic copepod was previously reported from *L. guentheri* and *L. suezensis* in the Mediterranean Sea and the Suez Canal, and other pufferfish species in the Indo-Pacific (mostly the genus *Takifugu* (Boxshall and El-Rashidy, 2009)). The prevalence was slightly lower than previously described for the Mediterranean Sea (34.7–80.0%) and similar to the Suez Canal (34.6%) (El-Lamie and Abdel-Mawla, 2012; Özak et al., 2012). Only *Lagocephalus* spp. were infected, while the sand-bound *T. flavimaculosus* (Farrag et al., 2016) either did not come into contact with the parasite or was no suitable host. The presence of *C. fugu* on *L. sceleratus* either presented a possible successful host change after its original immigration, or it was previously just unreported from this species. This study described *L. sceleratus* as a new host for *C. fugu*.

The copepod *Taeniacanthus lagocephali* was one of the common parasites of *L. guentheri*. The prevalence was similar to previous reports from the Mediterranean Sea (94%) and significantly higher than in the Suez Canal (34.6%). The presence of egg-carrying females confirmed the successful reproduction of this species in Israeli waters, as previously reported in Turkey and the Suez Canal (El-Lamie and Abdel-Mawla, 2012; Özak et al., 2012). The infection pathway was identical, based on the habitat preference, to *C. fugu* (Farrag et al., 2016).

### 4.3 Cestoda

All cestodes found were from specialized parasite families (Trypanorhyncha and Tetraphyllidea) with multiple-host life cycles. Pufferfish are particularly susceptible to trypanorhynch cestodes, showing a high diversity of these parasites in their native habitats, but also for other cestodes (Palm and Bray, 2014). The trypanorhynch cestode *Callitetrarhynchus gracilis* was among the satellite parasites and only present in larger specimens of *L. sceleratus* (L_T_ ≥ 45 cm). The scolex was well developed; distinguishing *L. sceleratus* as a third intermediate host according to Palm (2004). The cestode has been reported from over 140 intermediate hosts, including pufferfish (Palm, 2004; Beveridge et al., 2014). Small clupeids (sardines), engraulids (anchovies), or carangids (jacks) are second intermediate hosts, larger predatory fish are third intermediate hosts, and carcharinid sharks are the final hosts (Palm, 2004). Since large *L*. *sceleratus* fed on clupeids, carangids, and engraulids in the Mediterranean Sea, the infection pathway was accomplished by consumption of second intermediate hosts. *C. gracilis* has been globally reported, including in the Mediterranean and Red Sea (Palm, 2004; El-Sayed Abdou and Palm, 2008; Abdelsalam et al., 2016). This study identified *L. sceleratus* as a new intermediate host.

The trypanorhynch cestode *Nybelinia africana* was a rare parasite and only a single specimen was found in *L. guentheri*. The specimen had a fully developed scolex typical for plerocercoids in a third intermediate host (Palm, 2004). The second intermediate hosts are either small fish or euphausid shrimp (“krill”), a larger fish is the third intermediate host, and a large predatory fish or cephalopod is the fourth intermediate or paratenic host. The final hosts are a diverse group of elasmobranchs (Palm, 2004). Fish, including goatfish, and cephalopods were important prey items for *L. guentheri* in this study and are documented as intermediate hosts for this parasite (Palm, 2004). The genus *Nybelinia* Poche, 1926 is widely distributed and *N. africana* has been reported from the Mediterranean Sea, Atlantic, and the Indian Ocean surrounding Africa (Palm, 2004; Merella et al., 2010, 2016). The genus has previously been reported in *L. sceleratus* (Beveridge et al., 2014) and *N. africana* also infects the Lessepsian predatory fish *Fistularia comersonii* in the Mediterranean Sea, with a much higher prevalence (82.4–100.0%) (Merella et al., 2016). This study provided *L. guentheri* as a new host for *N. africana* and therefore was the first report for this species in a tetraodontid fish.

Larval Tetraphyllidea were either common or satellite species infecting *Lagocephalus* spp. Tetraphyllidea were reported to infect *L. guentheri* in the Suez Canal (El-Lamie and Abdel-Mawla, 2012), having a much lower prevalence than in the present study (prevalence of 6.1%). Several distinct morphotypes were present, possibly representing several species infecting the pufferfish. Tetraphyllidea have life cycles involving multiple hosts, including copepods, small fish, squid, and larger fish as paratenic hosts, with elasmobranchs as final hosts (Dick et al., 2006; Pardo-Gandarillas et al., 2009). They are reported globally, including the Mediterranean Sea (Keser et al., 2007; Pardo-Gandarillas et al., 2009; Felizardo et al., 2010). The high numbers of up to 200 plerocercoids in the pufferfish assigned them as paratenic hosts. The sand-bound *T. flavimaculosus* was not infected, so *Lagocephalus* spp. may have got infected in the *Posidonia oceanica* meadows they inhabit (Kalogirou et al., 2010, 2012; Kalogirou, 2013; Farrag et al., 2016). The comparatively higher prevalence and intensity of the infection in *L. guentheri* could be attributed to its earlier switch and a stronger emphasis on fish (several confirmed small intermediate Mediterranean hosts (Radujković and Šundić, 2014)) and cephalopods (suggested important intermediate hosts of Mediterranean shark tapeworms (Tedesco et al., 2020)) in its diet, thereby accumulating more plerocercoids over time. *L. sceleratus* and *L. suezensis* were new records for the Tetraphyllidea as paratenic hosts.

### 4.4 Nematoda

The nematodes belonged to the Raphisascarididae, maturing in teleost fish. *Hysterothylacium reliquens* was present in all four pufferfish species. It was one of the common or a secondary parasite. While adult nematodes were only present in *L. sceleratus* and one single specimen was found in *L. guentheri*, marking them as final hosts. The two smaller pufferfish species only acted as intermediate hosts. The entire life cycle and first hosts of *H. reliquens* are unknown, but larval stages are described in various fish, cephalopods, and decapod crustaceans (Deardorff and Overstreet, 1981). For the related *H. aduncum*, small crustaceans and polychaetes are known as the first and intermediate host (Køie, 1993; González, 1998). All pufferfish species in this study fed on crustaceans. The high prevalence and intensity in *L. sceleratus* could be attributed to the high percentage of crustaceans in its diet. *H. reliquens* is reported globally (Deardorff and Overstreet, 1981; Zhao et al., 2016), including the Mediterranean Sea (El-Sayed et al., 2004; Simsek et al., 2018). Reported hosts include pufferfish in the Atlantic Ocean (Rodríguez et al., 2019). Sequence analyses revealed high similarity (Query Cover 94–100% and identity 99.8–100%) with various vouchers from the Atlantic Ocean, Arabian Gulf and the Mediterranean Sea, confirming this nematode as globally distributed and not as a Lessepsian migrant species. This study identified *L. guentheri, L. sceleratus, L. suezensis*, and *T. flavimaculosus* as new hosts of *H. reliquens.*

The second nematode also belonged to the genus *Hysterothylacium*, being different from *H. reliquens*. The sequence analysis showed a high similarity to a *Hysterothylacium* sp. larval form reported from octopus (*Eledone* spp.) in the Mediterranean Sea with a similarly low prevalence (6.7%) by Guardone et al. (2020). The sole presence and low prevalence of larvae in *L. guentheri* could be attributed to the high percentage of cephalopods in its diet. *L. guentheri* thereby is a paratenic host for *Hysterothylacium* sp. Taxonomic status of this nematode as a native parasite to the Mediterranean Sea could also be confirmed by molecular markers. This study presented *L. guentheri* as a new host for this parasite and shed light on the life cycle of this not yet fully identified taxon.

The third nematode belonged to the genus *Raphidascaris* and was a rare parasite of *L. sceleratus*. The presence of an adult female specimen identified *L. sceleratus* as a final host of this parasite. *Raphidascaris* sp., *R. mediterraneus* and *R. macrouri*, were reported from predatory fish in the Mediterranean Sea (Dallarés et al., 2014; Pérez-i-García et al., 2015). Since the isolated parasite specimens lacked significant morphological features, identification to species level was impossible. The full life cycle of this genus is only known for one species, with invertebrates as the first hosts, with small fish as the secondary or paratenic hosts, and with large predatory fish as final hosts (Anderson, 2000). Pufferfish are known as final hosts to the genus *Raphidacaris* in the Pacific Ocean (Moravec and Justine, 2020). Therefore, the found *Raphidascaris* may theoretically be a Lessepsian migrant. This study demonstrated the second report of the genus *Raphidascaris* in Tetraodontidae and also described *L. sceleratus* as a new host.

### 4.5 Hirudinea

The marine leech *Trachellobdella lubrica* was a rare parasite of *L. sceleratus*. The species has a circumglobal distribution in warm and temperate waters, including the Arabian and the Mediterranean Sea (Saglam et al., 2003; Öktener and Utevsky, 2010), and prefers shallow waters (Noël and Sittler, 2021). *Trachelobdella lubrica* parasitizes on a wide range of hosts of all sizes (Epshtein, 1973) and has been reported to parasitize another Lessepsian migrant, the cornetfish *Fistularia commersonii* (Merella et al., 2016). This study identified *L. sceleratus* as a new host for this species and the first report of a *T. lubrica* parasitizing a member of the Tetraodontidae.

### 4.6 Overall parasite community

The comparison of our study with the available parasite assemblies of other pufferfish (both free-living and captive) showed some key differences. Monogenea, important ectoparasites of pufferfish in the Indo-Pacific Ocean, were conspicuously absent and the diversity of Digenea was also significantly lower (Ogawa and Inouye, 1997; Ogawa and Yokoyama, 1998; Fajer-Ávila et al., 2004; Álvarez-Borrego and Fajer-Ávila, 2006; Prasad and Radhakrishnan, 2010). The same differences occurred in the data from Hawaii (Palm and Bray, 2014), where Monogenea were also rare, but the Digenea were much more diverse. Of the studied species, only *L. sceleratus* has been known to be infected by Monogenea in Chinese waters (Jianying et al., 2003; Hung et al., 2020). Monogenea belong to the most successful Lessepsian parasites (Paperna, 1972; Ktari and Ktari, 1974; Pasternak et al., 2007). Their absence in pufferfish from the Mediterranean Sea suggests either a successful strip off by the Suez Canal passage, or their general lack in the Red Sea, from which no records were available. The absence of Digenea (except for *M. dayawanensis*) could be explained by the need for intermediate hosts for establishment in a new habitat, which is not present in the Mediterranean Sea. Compared to the studies conducted in the Mediterranean Sea and Suez Canal (El-Lamie and Abdel-Mawla, 2012; Özak et al., 2012; Bakopoulos et al., 2017), the diversity of native parasites was much higher, while the Lessepsian copepods could be confirmed. In comparison to Hawaii (Palm and Bray 2014), a loss of parasite diversity for the whole family of Tetraodontidae in the Mediterranean Sea is also suggested by the E/E ratio differences of this study. The combined E/E ratio was higher (0.5 vs. 0.11) (Palm and Bray, 2014), indicating the loss of endoparasites compared to the Indo-Pacific. At the species level, however, compared to the studied Hawaiian members of the same pufferfish genera (*Lagocephalus lagocephalus* (L.) and *Torquigener hypselogeneion* (Bleeker, 1852)), the number of found parasite species per pufferfish species was higher (2–7 vs. 2–3) in average, while the E/E ratios per species were similar (0–0.67 vs. 0–1), typical for a diverse ecosystem (Rückert et al., 2009). The shared parasites between the Mediterranean Sea and Hawaii were copepods, most likely specialists, and generalistic Rhaphidascarididae nematodes of the genus *Hysterothylacium* Ward & Magath, 1917. These shared parasites are further supporting the strip off of native parasites during the transit of the Suez Canal, except for very few oioxenic species, and the acquisition of a rich fauna of generalists in the Mediterranean Sea. Differences in parasite communities between the four pufferfish species could be explained by their different habitat preferences and prey spectrum (Kalogirou et al., 2010, 2012; Kalogirou, 2013; Farrag et al., 2016). As Fig. 2 illustrates, *L. guentheri* was separated from the others. Although, the general ecology of the investigated pufferfish species in the Mediterranean Sea is unknown, *L. guentheri* is known to be habitat changing and migratory. Additionally, it had the most diverse invertebrate diet and five different teleost prey taxa were found. The other three pufferfish species showed an overlapping pattern, these taxa are known to be less migratory and showed lower variety in invertebrate prey taxa.

The absence of *Anisakis* sp., a potentially zoonotic nematode, could be explained by unsuitable conditions for the first host in the Levantine Basin, despite its presence in the rest of the Mediterranean Sea (Anderson, 2000; Mayzaud et al., 2000) and the difference in identified *Hysterothylacium* species by previously neglected molecular identification.

This study has significantly increased our knowledge of the parasite assemblages of four Lessepsian pufferfish in the Mediterranean Sea, on both local and global levels. We found three species of oioxenic Lessepsian parasites. Two copepods could be confirmed from previous studies (El-Lamie and Abdel-Mawla, 2012; Özak et al., 2012). The Digenea *M. dayawanensis* is a new, previously unreported, Lessepsian endoparasite and has possibly become established in the Mediterranean Sea by now. The findings of this study showed similarities to the parasite community of the cornetfish *F. commersonii*, another large Lessepsian predatory fish, including shared parasites (Merella et al., 2016). Both pufferfish and cornetfish lost most or all of their presumed ectoparasites (enemy release hypothesis) from the Red Sea, and retained endoparasites during the transit through the Suez Canal, which can be considered one factor for their success as alien species (Arndt and Schembri, 2015; Filiz et al., 2017). Pufferfish and cornetfish (Merella et al., 2016), in turn, also acquired a diverse new assembly of native Mediterranean, mostly endoparasitic, generalists.

As the fishes we sampled at availability by different sampling methods, sites and seasons, no spatial nor temporal analysis could be conducted. However, as visibile in Table 3, a host species sampled twice in winter shows the highest parasite species richness, and another host species that was sampled eight times (within nine month) hosted seven parasite species. Therefore, no further seasonal interpretation of found parasite community seems to be feasible.

All studies conducted on the parasites of Lessepsian pufferfish, including this one, are so far limited to the Southern Mediterranean Sea (Levantine and the Aegean Sea) and the Suez Canal (El-Lamie and Abdel-Mawla, 2012; Özak et al., 2012; Bakopoulos et al., 2017). Pufferfish, especially *L. sceleratus*, however, have extended their range to the western parts of the Mediterranean Sea and into the Black Sea (Golani et al., 2021). While neither this study nor the others could find any transfer of possible zoonotic parasites to commercially important species or the introduction of dangerous Lessepsian parasites, this issue should be addressed for the rest of the Mediterranean Sea. Particular emphasis should be placed upon a possible ongoing co-invasion of pufferfish and parasites through the Suez Canal, to detect potentially dangerous species. Additional research on the parasites of juvenile pufferfish would help to better understand their ecological niche and competition with native species. Finally, research on the rare spiny blaasop (*Tylerius spinosissimus*) could shed more light on the little-known deepwater parasite communities and possible Lessepsian deepwater parasites. Due to the irregular sampling, the sample size in this study varied between years and locality, and many specimens were examined after being frozen for some time. Therefore, it was possible that some parasites (ectoparasites) were displaced or migrated during transportation or preservation, as was the case for *Trachelobdella lubrica*. Pufferfish in the Mediterranean Sea are not commercially fished, so opportunistic sampling was the only approach that could be adopted for the study, as it had been done before for other non-commercial marine species with decent results (Santoro et al., 2010; Aznar et al., 2012: Merella et al., 2016).

## Declaration of competing interest

None.
